# Lacosamide-induced sinus node dysfunction followed by severe agranulocytosis

**DOI:** 10.1186/s12883-021-02253-1

**Published:** 2021-06-08

**Authors:** Makoto Shibata, Reona Hoshino, Chisato Shimizu, Masayuki Sato, Natsumi Furuta, Yoshio Ikeda

**Affiliations:** 1 Department of Neurology, Takasaki General Medical Center, National Hospital Organization, 36 Takamatsu-cho, Takasaki, 370-0829 Gunma Japan; 2 Department of Neurology, Gunma University Graduate School of Medicine, 3-39-22 Showa-machi, Maebashi, 371-8511 Gunma Japan

**Keywords:** Lacosamide, Neutropenia, Agranulocytosis, Sinus node dysfunction, Adverse effects

## Abstract

**Background:**

Lacosamide (LCM) is the antiepileptic drug approved by the U.S. Food and Drug Administration in 2008 that facilitates slow activation of the voltage-gated sodium channels. Neutropenia and cardiac events including sinus node dysfunction (SND) and atrioventricular block have been previously reported as adverse effects of LCM. To date, there have been no reports of severe agranulocytosis resulting in death associated with LCM. Additionally, there have been no reports of concomitant SND and agranulocytosis after LCM administration. Herein we report the first case of LCM-induced severe SND followed by agranulocytosis.

**Case presentation:**

The patient with focal epilepsy was initiated on LCM 100 mg/day and the dose was increased to 200 mg/day on the 9^th^ hospital day. Severe SND developed on the 10^th^ hospital day and LCM was discontinued. Thereafter agranulocytosis appeared on the 11^th^ hospital day, and the patient died from septic shock on the 15^th^ hospital day.

**Conclusions:**

This case illustrates the need for careful follow-up of the electrocardiogram and the complete blood cell counts when initiating LCM. Moreover, it should be noticed that various side effects may occur simultaneously in the early period of LCM use, even for a short time and at low dosages.

## Background

Lacosamide (LCM) is one of the well-tolerated anti-epileptic drugs (AEDs) that facilitates slow activation of voltage-gated sodium channels [1, 2]. It has been noted that LCM can cause adverse effects (AEs) similar to those of conventional sodium channel blockers (SCBs). Although rare, there are increasing reports of AEs associated with LCM [3].

To date, there have been several reports of neutropenia as common AEs of LCM; [4–8] however, there are no reports of severe agranulocytosis resulting in death. Additionally, several cases of sinus node dysfunction (SND) and atrioventricular (AV) block have been reported as serious AEs of LCM, [9–11] however, there have been no reports of concomitant SND and agranulocytosis.

Herein we report a case of LCM-induced SND followed by agranulocytosis resulting in death from septic shock. Although concomitant occurrence of SND and agranulocytosis due to LCM is incidental and extremely rare, clinicians should be aware of the respective severe AEs.

## Case presentation

A 78-year-old Japanese female was diagnosed with focal epilepsy two years prior to admission based on repeated right hemiconvulsion evolving into generalized tonic–clonic seizure with an electroencephalography finding of frequent sharp waves in the left parietal lobe. Her status was stable on treatment with levetiracetam (LEV) 2000 mg/day orally, however, episodic disturbance of consciousness appeared and gradually worsened one month prior to admission. Phenytoin (PHT) 200 mg/day was given in conjunction with LEV at another hospital 16 days prior to admission. Nevertheless, there was little improvement in the frequency of impaired consciousness, and she was transferred to our hospital to adjust AEDs.

**Fig. 1:**
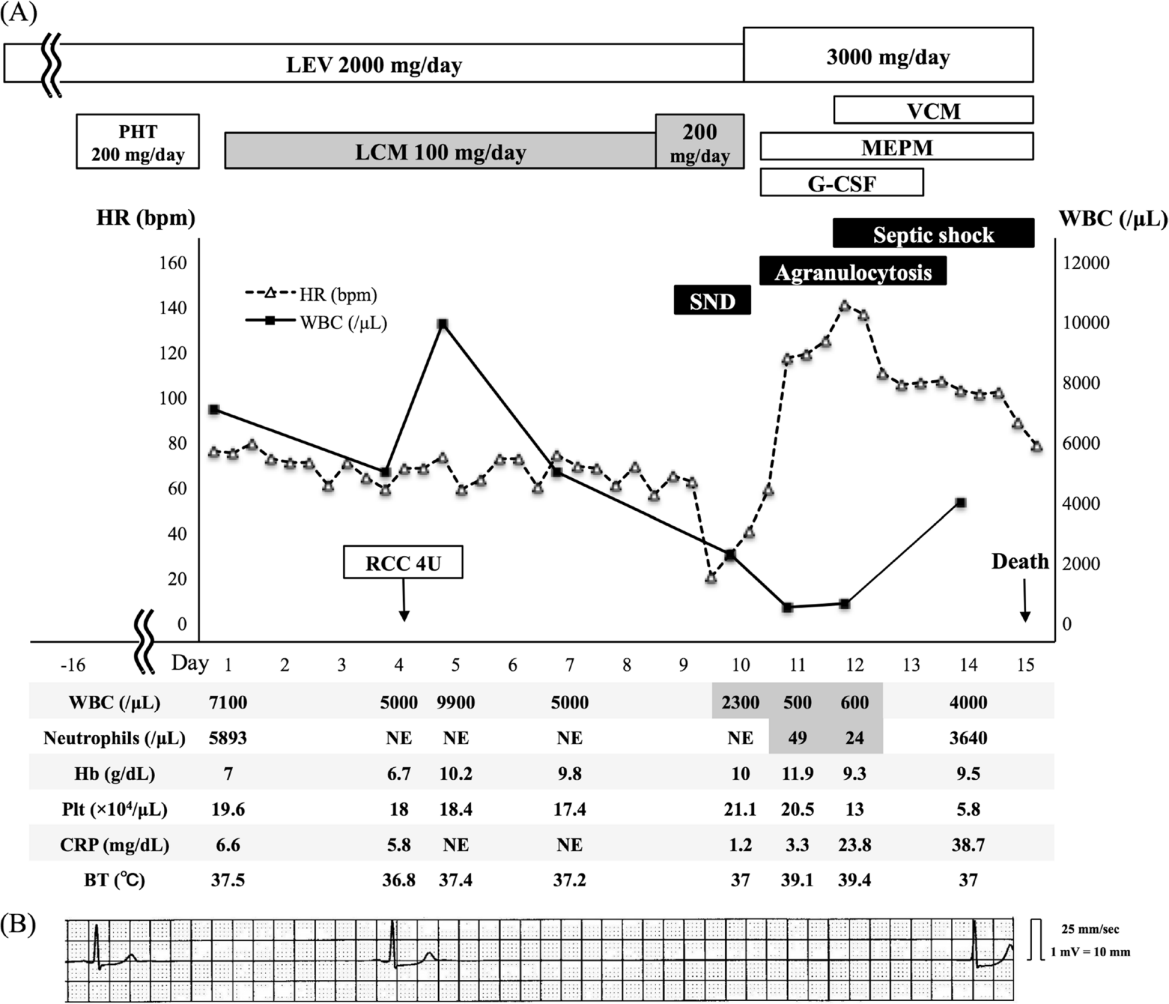
Clinical course, laboratory, and electrocardiogram findings of the present case. The patient’s clinical course and the chronological changes of HR and WBC counts (upper graph, **A**), laboratory results of WBC, neutrophils, Hb, Plt, CRP, and BT (lower table, **A**) are shown. An electrocardiogram finding on the 10^th^ hospital day is also shown with a maximum sinus arrest of 5.6 s (**B**). Abbreviations: BT = body temperature; CRP = C-reactive protein; G-CSF = granulocyte-colony stimulating factor; Hb = hemoglobin; HR = heart rate; LCM = lacosamide; LEV = levetiracetam; MEPM = meropenem; NE = not examined; PHT = phenytoin; Plt = platelet; RCC = red blood cell concentrates; SND = sinus node dysfunction; VCM = vancomycin; WBC = white blood cell

At the time of admission, her level of consciousness was alert and there was no obvious evidence of epileptic seizures. Physical examination on admission revealed blood pressure (BP) 106/52 mm Hg, heart rate (HR) 76 beats per minute (bpm), body temperature (BT) 37.5 °C, and respiratory rate (RR) 22 breaths per minute, and the electrocardiogram (ECG) showed no arrhythmia and PR interval was normal (0.16 s). Her height, body weight, and body mass index (BMI) were 152 cm, 42 kg, and 18.2 kg/m^2^, respectively. Figure 1A shows the clinical course and the chronological laboratory findings of the patient. Her complete blood cell counts (CBCs) showed total white blood cell (WBC) counts of 7100 /μL with neutrophil counts of 5893 /μL, hemoglobin level of 7.0 mg/dL, red blood cell counts of 413 × 10^4^ /μL, mean corpuscular volume of 63.7 fl, mean corpuscular hemoglobin concentration of 26.6%, and platelet counts of 19.6 × 10^4^ /μL, indicating hypochromic microcytic anemia due to iron deficiency. Her blood chemistry tests showed normal liver and renal function: aspartate aminotransferase (AST) of 13 U/L, alanine aminotransferase (ALT) of 8 U/L, blood urea nitrogen (BUN) of 15.1 mg/dL, and creatinine (Cr) of 0.34 mg/dL. Her serum C-reactive protein (CRP) was mildly elevated to 6.6 mg/dL and chest computed tomography showed mild aspiration pneumonia in the left S10 segment of left lung. Since she was in good condition without decreased percutaneous oxygen saturation (SpO_2_), antibiotics were not administered. LEV 2000 mg/day was continued orally and PHT was discontinued, thereafter, the patient was initiated on LCM at 100 mg/day orally because the patient did not accept to increase the LEV dose due to drug-induced drowsiness. The stool occult blood on admission was positive, but the patient did not agree to undergo a gastrointestinal endoscopy, thereafter, the patient received a blood transfusion containing 4 units of red blood cell concentrates (RCC) on the 4^th^ hospital day to treat anemia.

The dose of LCM was increased to 200 mg/day on the 9^th^ hospital day, as per the Japanese guidelines for maintenance doses, and subsequently on the 10^th^ hospital day, a sinus bradycardia with HR of 20 bpm suddenly began to appear, with a maximum sinus arrest of 5.6 s (Fig. 1B). The PR interval was 0.16 s and did not change during her hospitalization. There was no decrease in BP or no symptomatic syncope due to SND, while WBC counts were decreased to 2300/μL on the 10^th^ hospital day. The additional blood chemistry tests showed AST of 16 U/L, ALT of 10 U/L, BUN of 15.8 mg/dL, Cr of 0.33 mg/dL, and potassium of 4.45 mmol/L. Drug-induced SND and leukopenia were suspected and LCM was immediately discontinued. The bradycardia gradually improved until the next day with HR of 59 bpm. There was no evidence of sepsis causing neutropenia at this point, and CRP was decreased to 1.2 mg/dL.

On the 11^th^ hospital day, a decrease in SpO_2_ requiring high-flow oxygen appeared. An analysis of arterial blood (O_2_ 10 L/min via non-rebreather mask) showed a pH of 7.49, a partial pressure of carbon dioxide of 31.9 mm Hg, and a partial pressure of oxygen of 72.3 mm Hg. Although serum N-terminal pro-brain natriuretic peptide level was elevated to 1320 pg/mL (reference range < 125 pg/mL), a transthoracic echocardiogram showed a normal ejection fraction of the left ventricle (63%) without signs of asynergy and pulmonary hypertension, and normal inferior vena cava diameter (13 mm) with normal inspiratory collapse. A chest X-ray showed an infiltrative shadow in the left lung field, which was suspected to be exacerbation of the pneumonia recognized at admission. The WBC counts decreased to 500/μL and the neutrophil counts to 49/μL, leading to a diagnosis of drug-induced agranulocytosis. Fever of over 39 °C appeared. After blood, sputum, and urine cultures were submitted, meropenem (3 g/day) and granulocyte-colony stimulating factor (G-CSF) (100 μg/day) were administered. No bacterial growth was confirmed in the blood and urine cultures, while the sputum cultures showed a growth of *Acinetobacter baumannii/calcoaceticus complex* and *Klebsiella pneumoniae* that were sensitive to carbapenem antibiotics. Since LCM was discontinued, LEV was replaced from oral to intravenous administration, and the dose was increased to 3000 mg/day in order to reinforce the treatment of epilepsy.

On the 12^th^ hospital day, the vital signs were worsened to BP 69/49 mm Hg, HR 123 bpm, BT 39.4 °C, RR 34 breaths per minute, and she became unresponsive to calls. Consequently, fluid resuscitation was started, and vancomycin was administered with dose adjustment based on therapeutic drug monitoring (TDM). Since hypotension did not improve even with adequate fluid resuscitation, norepinephrine was started and increased to maintain mean arterial pressure at 65 mm Hg. The WBC counts were 600/μL and the neutrophil counts were 24/μL with still no improvement in agranulocytosis, and serum procalcitonin level was markedly elevated at 25.55 ng/mL (reference range < 0.50 ng/mL), suggesting severe sepsis. Generally, 28–49% of severe sepsis cases are known to show negative cultures [12] Although the blood cultures of the patient were negative, septic shock was strongly suspected due to fever associated with agranulocytosis, hypotension unresponsive to adequate rehydration, tachypnea, disturbance of consciousness, and the absence of other diseases causing hypotension.

On the 14^th^ hospital day, the patient’s WBC and neutrophil counts improved to 4000/μL and 3640/μL, respectively, therefore, G-CSF administration was discontinued. Although the SND and agranulocytosis improved, the patient could not recover from septic shock, and died on the 15^th^ hospital day.

## Discussion and conclusion

The present case represented two clinically important points. First, LCM can cause severe agranulocytosis leading to death. Second, LCM can cause both agranulocytosis and SND simultaneously, therefore, careful follow-up of CBCs and ECG are required especially in the early period of treatment.

Since the patient received no additional medications other than LCM, and there was a rapid improvement of neutropenia after LCM discontinuation and G-CSF administration, LCM-induced neutropenia was strongly suspected. The Naranjo adverse drug reaction probability scale was 8 points, [13] indicating that LCM was a “probable” cause of agranulocytosis. Table 1 shows a summary of previous reports on LCM-induced neutropenia [4–8]. Agranulocytosis is diagnosed as a condition in which the number of neutrophils in the peripheral blood is less than 500/μL, and its cause is usually drug-induced [14, 15]. To date, only a single case of agranulocytosis due to LCM has been reported (Table 1) [6]. To our best knowledge, this is the first case of LCM-induced severe agranulocytosis leading to death due to septic shock. Among all AEDs, SCBs such as carbamazepine (CBZ) and PHT are known to cause agranulocytosis, [16] and CBZ had the highest potential for agranulocytosis [17]. LCM is a commonly used AED among SCBs, therefore, it is important to clarify which patients are at high risk for LCM-induced agranulocytosis as a future direction.

**Table 1:** Summary of previous reports on lacosamide-induced neutropenia

Author (year)	Number of patients	Age, Sex	LCM dosage	LCM duration	Other AEDs	Adverse event	Clinical outcome (Days to remission)
Husain et al. (2012)[4]	2	ND	ND	ND	ND	**Neutropenia**	ND
Novy et al. (2013)[5]	7(^a^)	ND	ND	ND	ND	**Neutropenia** or Rash (^a^)	ND
Zadeh et al. (2015)[6]	1	25, M	400 mg/day	46 days	LTG, LEV (Dose unknown)	**Agranulocytosis**	**Improved ** (7 days after LCM was discontinued)
Welsh et al. (2017)[7]	6	ND (children)	ND	ND	ND	**Neutropenia**	ND
Rao et al. (2018)[8]	1	20, M	200 mg/day	10 days	VPA 1000 mg/day, CLB 10 mg/day	**Neutropenia**	**Improved ** (2 days after LCM was discontinued)
Present case	1	80, F	200 mg/day	9 days	LEV 2000 mg/day	**Agranulocytosis**	**Death from sepsis**

*Abbreviations*: *ND* Not described, *M* Male, *F* Female, *LCM* Lacosamide, *AED* Anti-epileptic drug, *LTG* Lamotrigine, *LEV* Levetiracetam, *VPA* Valproic acid, *CLB* Clobazam

(^a^) The number of events is the sum of allergic events including neutropenia or rash

It is also important to note that agranulocytosis in the present case occurred at a low LCM dose of 200 mg/day. Previous reports have shown that the increased risk of drug-induced neutropenia is more pronounced in the first month of treatment and is not related to the daily dosage [18]. Careful follow-up of CBCs is required especially in the first month after initiation of LCM even at low doses. Additionally, it is important to discontinue LCM as soon as any suspicious signs appear.

It is especially important to note that LCM can cause agranulocytosis and SND simultaneously. LCM is known to cause various cardiac conduction disorders (CCDs), including dose-dependent prolongation of the PR interval, [19] second-degree AV block, [10] third-degree AV block, [11] and SND [9]. Previous reports of CCDs due to LCM are more common in patients with concomitant use of SCBs or drugs that prolong the PR interval [9, 10]. In this case, PHT was used until the time of admission, and was discontinued promptly after admission. Although there was no detailed evaluation of PHT blood levels, the recent use of PHT may have played a role in the development of SND. The risk of CCDs associated with LCM remains unknown except for concomitant medications. This patient had no prolongation of PR interval (0.16 s), sinus bradycardia (HR = 76 bpm), renal dysfunction, and hepatic dysfunction on admission. On the other hand, she was underweight (BMI = 18.2 kg/m^2^) and elderly, which may have affected her drug metabolism. Further evaluation of the details of LCM-induced CCDs risk factors is needed as more cases are accumulated.

We need to investigate whether the bradycardia in this patient was ictal bradycardia syndrome (IBS), but we believe it is likely to be LCM-induced bradycardia. First, the patient’s consciousness was alert at the time of onset of SND, and there were no findings suggestive of epilepsy. Second, the duration of IBS was previously reported to be an average of 23.2 s (range, 4–36 s), [20]. which was quite different from our case showing the bradycardia persisted for over 24 h.

There are several limitations in this case report. First, the patient was transfused with RCC before the appearance of SND and agranulocytosis, and the association between RCC transfusion and these rare complications cannot be excluded. However, the RCC transfusion was performed on the 4th hospital day, and SND and agranulocytosis appeared on the 10th and 11th hospital days, respectively. Considering the time lag of each event, it was unlikely that any of the complications were related to the RCC transfusion. Although the transfusion-related neutropenia has been reported as a rare side effect, it usually occurs within a few hours after transfusion and was not consistent with the present case [21]. Second, the blood concentration of LCM was not measured in this case. Under normal conditions, LCM is a drug that is not required a strict TDM like other anticonvulsants such as PHT or CBZ [22]. In this case, there was no severe renal dysfunction or drug interactions that could have increased LCM blood levels. Considering the dosage and duration of LCM administration, it is unlikely that the LCM blood levels were significantly elevated above the therapeutic range.

In conclusion, this report revealed that LCM, even used in low doses, can cause serious AEs including agranulocytosis and cardiac events such as SND. Thus, careful ECG and CBCs follow-up are required, especially in the first month after initiation of treatment. Additionally, if one AE associated with the use of AEDs occurs, clinicians should evaluate whether other AEs have also emerged or not.

## Data Availability

Not applicable.

## Abbreviations

AEDs: Anti-epileptic drugs
AEs: Adverse effects
ALT: Alanine aminotransferase
AST: Aspartate aminotransferase
AV: Atrioventricular
BMI: Body mass index
BP: Blood pressure
bpm: Beats per minute
BT: Body temperature
BUN: Blood urea nitrogen
CBCs: Complete blood cell counts
CBZ: Carbamazepine
CCDs: Cardiac conduction disorders
Cr: Creatinine
CRP: C-reactive protein
ECG: Electrocardiogram
G-CSF: Granulocyte-colony stimulating factor
HR: Heart rate
IBS: Ictal bradycardia syndrome
LCM: Lacosamide
LEV: Levetiracetam
PHT: Phenytoin
RCC: Red blood cell concentrates
RR: Respiratory rate
SCBs: Sodium channel blockers
SND: Sinus node dysfunction
SpO_2_: Percutaneous oxygen saturation
TDM: Therapeutic drug monitoring
WBC: White blood cell

## References

[1] Strzelczyk A, Zöllner JP, Willems LM, Jost J, Paule E, Schubert-Bast S (2017). Lacosamide in status epilepticus: Systematic review of current evidence. Epilepsia.

[2] Errington AC, Stöhr T, Heers C, Lees G (2008). The investigational anticonvulsant lacosamide selectively enhances slow inactivation of voltage-gated sodium channels. Mol Pharmacol.

[3] Li J, Sun M, Wang X (2020). The adverse-effect profile of lacosamide. Expert Opin Drug Saf.

[4] Husain A, Chung S, Faught E, Isojarvi J, McShea C, Doty P (2012). Long-term safety and efficacy in patients with uncontrolled partial-onset seizures treated with adjunctive lacosamide: results from a Phase III open-label extension trial. Epilepsia.

[5] Novy J, Bartolini E, Bell GS, Duncan JS, Sander JW (2013). Long-term retention of lacosamide in a large cohort of people with medically refractory epilepsy: a single centre evaluation. Epilepsy Res.

[6] Zadeh WW, Escartin A, Byrnes W, Tennigkeit F, Borghs S, Li T (2015). Efficacy and safety of lacosamide as first add-on or later adjunctive treatment for uncontrolled partial-onset seizures: a multicentre open-label trial. Seizure.

[7] Welsh SS, Lin N, Topjian AA, Abend NS (2017). Safety of intravenous lacosamide in critically ill children. Seizure.

[8] Rao NP, Sheth S, Varambally S (2018). Lacosamide Precipitated neutropenia in a patient with bipolar disorder and comorbid epilepsy. Indian J Psychol Med.

[9] Chinnasami S, Rathore C, Duncan JS (2013). Sinus node dysfunction: an adverse effect of lacosamide. Epilepsia.

[10] Nizam A, Mylavarapu K, Thomas D, Briskin K, Wu B, Saluja D (2011). Lacosamide-induced second-degree atrioventricular block in a patient with partial epilepsy. Epilepsia.

[11] Krause LU, Brodowski KO, Kellinghaus C (2011). Atrioventricular block following lacosamide intoxication. Epilepsy Behav.

[12] Gupta S, Sakhuja A, Kumar G, McGrath E, Nanchal RS, Kashani KB (2016). Culture-negative severe sepsis: nationwide trends and outcomes. Chest.

[13] Naranjo CA, Busto U, Sellers EM, Sandor P, Ruiz I, Roberts EA (1981). A method for estimating the probability of adverse drug reactions. Clin Pharmacol Ther.

[14] Andrès E, Maloisel F (2008). Idiosyncratic drug-induced agranulocytosis or acute neutropenia. Curr Opin Hematol.

[15] Andersohn F, Konzen C, Garbe E (2007). Systematic review: agranulocytosis induced by nonchemotherapy drugs. Ann Intern Med.

[16] Ibáñez L, Vidal X, Ballarín E, Laporte JR (2005). Population-based drug-induced agranulocytosis. Arch Intern Med.

[17] Perucca P, Gilliam FG (2012). Adverse effects of antiepileptic drugs. Lancet Neurol.

[18] van Staa TP, Boulton F, Cooper C, Hagenbeek A, Inskip H, Leufkens HG (2003). Neutropenia and agranulocytosis in England and Wales: incidence and risk factors. Am J Hematol.

[19] Ben-Menachem E, Biton V, Jatuzis D, Abou-Khalil B, Doty P, Rudd GD (2007). Efficacy and safety of oral lacosamide as adjunctive therapy in adults with partial-onset seizures. Epilepsia.

[20] Britton JW, Ghearing GR, Benarroch EE, Cascino GD (2006). The ictal bradycardia syndrome: localization and lateralization. Epilepsia.

[21] Hauck-Dlimi B, Ruppel R, Zimmermann R, Strobel J, Reil A, Eckstein R (2016). Transfusion-related alloimmune neutropenia with no pulmonary complications: one donor-five cases. Transfusion.

[22] Schultz L, Mahmoud SH (2020). Is therapeutic drug monitoring of lacosamide needed in patients with seizures and epilepsy?. Eur J Drug Metab Pharmacokinet.

